# Association Between Epilepsy and Leigh Syndrome With *MT-ND3* Mutation, Particularly the m.10191T>C Point Mutation

**DOI:** 10.3389/fneur.2021.752467

**Published:** 2021-12-10

**Authors:** Ji-Hoon Na, Min Jung Lee, Chul Ho Lee, Young-Mock Lee

**Affiliations:** ^1^Department of Pediatrics, Yonsei University College of Medicine, Seoul, South Korea; ^2^Epilepsy Research Institute, Yonsei University College of Medicine, Seoul, South Korea

**Keywords:** mitochondrial DNA-associated Leigh syndrome, MT-ND3, m10191T>C, epilepsy, Lennox-Gastaut syndrome

## Abstract

**Background and Purpose:** Recent advances in molecular genetic testing have led to a rapid increase in the understanding of the genetics of Leigh syndrome. Several studies have suggested that Leigh syndrome with *MT-ND3* mutation is strongly associated with epilepsy. This study focused on the epilepsy-related characteristics of Leigh syndrome with *MT-ND3* mutation identified in a single tertiary hospital in South Korea.

**Methods:** We selected 31 patients with mitochondrial DNA (mtDNA) mutations who were genetically diagnosed with mtDNA-associated Leigh syndrome. Among them, seven patients with *MT-ND3* mutations were detected. We reviewed various clinical findings such as laboratory findings, brain images, electroencephalography data, seizure types, seizure frequency, antiepileptic drug use history, and current seizure status.

**Results:** The nucleotide changes in the seven patients with the Leigh syndrome with *MT-ND3* mutation were divided into two groups: m.10191T>C and m.10158T>C. Six of the seven patients were found to have the m.10191T>C mutations. The median value of the mutant load was 82.5%, ranging from 57.9 to 93.6%. No particular tendency was observed for the first symptom or seizure onset or mutant load. The six patients with the m.10191T>C mutation were diagnosed with epilepsy. Three of these patients were diagnosed with Lennox–Gastaut syndrome (LGS).

**Conclusion:** We reported a very strong association between epilepsy and *MT-ND3* mutation in Leigh syndrome, particularly the m.10191T>C mutation. The possibility of an association between the epilepsy phenotype of the m.10191T>C mutation and LGS was noted.

## Introduction

Leigh syndrome (or subacute necrotizing encephalomyelopathy) is the most common clinical syndrome of mitochondrial disease that occurs in the pediatric patients. Leigh syndrome is further divided into mitochondrial DNA (mtDNA) associated and nuclear associated (1–4). Recent advances in molecular genetic testing approaches have led to a rapid increase in the understanding of the genetics of Leigh syndrome. Due to this comprehensive genomic testing, a genotype–phenotype correlation has been actively attempted according to the genetic mutations of Leigh syndrome. In particular, the genotype–phenotype correlation of mtDNA-associated Leigh syndrome is more challenging, as heteroplasmy must be considered and genetic mutation type (2–5).

In total, mutations in 14 mitochondrial genes are known to be associated with Leigh syndrome (*MT-ATP6, MT-CO3, MT-ND1, MT-ND2, MT-ND3, MT-ND4, MT-ND5, MT-ND6, MT-T1, MT-TK, MT-TL1, MT-TL2, MT-TV*, and *MT-TW*) (4). Genotype–phenotype studies of each of these mutations are currently underway. Among them, several studies have found that Leigh syndrome with *MT-ND3* mutation (hereafter referred as Leigh syndrome with *MT-ND3* mutation) is strongly associated with epilepsy (6–8). This knowledge may assist neurologists with the development of treatment plans and prognosis predictions for the patients with Leigh syndrome with the *MT-ND3* mutation.

Therefore, in this study, we focused on the epilepsy-related characteristics of the cases of Leigh syndrome with *MT-ND3* mutation identified in a single tertiary hospital in Korea.

## Methods

### Selection of the Patients

Patients with mtDNA mutations who were clinically diagnosed with Leigh syndrome between 2006 and 2018 at the Mitochondrial Disease Clinic of Gangnam Severance Hospital in the Republic of Korea were selected. The clinical diagnosis of Leigh syndrome was based on stringent diagnostic criteria described by Rahman et al. (2–4). The criteria is outlined as follows: presentation of a progressive neurological disease with symptoms of motor and intellectual developmental delays, signs and symptoms of brainstem and/or basal ganglia disease, raised lactate concentrations in the blood and/or cerebrospinal fluid (CSF), and bilateral symmetric hyperintense signal abnormality in the brainstem and/or basal ganglia shown on T2-weighted images in MRI of the brain. Sequencing of whole mtDNA was performed in all the patients clinically diagnosed with Leigh syndrome using next-generation sequencing (NGS) technology. Among them, 31 patients were genetically diagnosed with mtDNA-associated Leigh syndrome and among these, 7 patients with *MT-ND3* mutations were detected. Each parent received a detailed explanation of this study and signed an informed consent form before their child participated in this study. This study was approved by the Institutional Review Board of Gangnam Severance Hospital, Yonsei University College of Medicine (3-2017-0168).

### Phenotype Analysis

#### Clinical Manifestations

The clinical manifestations of Leigh syndrome with *MT-ND3* mutation included in this study were reviewed. The first symptoms at disease onset and organ involvement at the time of the last follow-up were investigated as general characteristics of the patients. Patients were divided into two groups according to the criteria of early-onset Leigh syndrome: (1) <24 months old and (2) >24 months old when the first symptom occurred. The first symptom at disease onset was used to identify the most dominant symptom in the patients. For organ involvement at the time of the last follow-up, all the organs involved in Leigh syndrome, including the central nervous system (CNS), muscles, respiratory system, gastrointestinal system (e.g., gastroesophageal reflux disease, motility disorders), endocrine system, kidneys (e.g., tubular acidosis), eyes (e.g., optic atrophy, ptosis), ears, and skeletal system, were checked.

The prevalence of epilepsy was high in the patients with Leigh syndrome with *MT-ND3* mutation. Therefore, the epilepsy-related characteristics of all the seven patients were examined and have been described in detail including seizure types, seizure frequency, electroencephalography (EEG) findings, antiepileptic drug history, and current seizure status.

#### Laboratory Findings

The plasma lactate levels of the patients were obtained and defined as mild, moderate, or severe, if they were higher than the normal reference value by at least 2-fold, 3-fold, or 4-fold, respectively (9, 10). These values were evaluated as sequential variables ranging from one to four. Muscle biopsies were also performed on the patients and observed under light and electron microscopes. Light microscopy revealed specific findings for mitochondrial diseases such as ragged red fibers and electron microscopy revealed pleoconia and megaconia. In addition, muscle biopsy samples were processed using routine immunohistochemical staining (9).

#### Magnetic Resonance Imaging of the Brain and Proton Magnetic Resonance Spectroscopy

Mitochondrial DNA-associated Leigh syndrome is characterized by bilateral symmetric hyperintense signal abnormalities in the brainstem and/or basal ganglia observed on T2-weighted MRIs (4). The MRI findings of the patients were obtained and separated according to the affected area such as the basal ganglia, brainstem, and thalamus. MRS can also be useful in detecting lactate peaks in the brain (11). Therefore, MRS abnormalities were obtained to establish the diagnosis.

### Genotype Analysis

#### Whole Mitochondrial Gene Sequence Analysis Using NGS Technology

Pathogenic mutations, including point mutations and large deletions, can occur in the mitochondrial genome. Therefore, the diagnosis of mtDNA-related Leigh syndrome should include the detection and quantification of sequence changes at any position of the mitochondrial genome (12). Whole mitochondrial gene sequence analysis was performed on all the patients who were clinically diagnosed with Leigh syndrome in this study. NGS technology was used to genetically confirm mtDNA Leigh syndrome and quantify the heteroplasmic mutant load of mtDNA. The sequence results were compared with the human mitochondrial reference (GenBank ID: NC_012920.1) (12–14).

#### Sample Preparation

Deoxyribonucleic acid was extracted from peripheral blood leukocytes using the QIAcube System and the QIAamp DNA Blood Mini Extraction Kit (Qiagen, Hilden, Germany, UK) and stored in 10 mM Tris buffer solution at −20°C. mtDNA was amplified using a long-range PCR. PCR reaction conditions were 98°C for 30 s, 30 cycles of 98°C for 10 s, 72°C for 8 min 15 s, and a final extension at 72°C for 10 min. PCR products were run on a 1% agarose gel and the expected 16.5 Kb fragments were excised. DNA was purified using Agencourt AMPure XP (Beckman Coulter, Brea, California, USA). Quantification was performed using the 4,200 TapeStation (Agilent Technologies, Santa Clara, California, USA) (15).

#### Library Preparation and Sequencing

The PCR product was fragmented into 150–200 base pair (bp) segments with a NEBNext dsDNA Fragmentase^®^ (New England Biolabs, Ipswich, Massachusetts, USA), according to the protocol of the manufacturer. The enzyme-fragmented PCR product was used as an input to the Accel-NGS^®^ 2S PCR-free DNA Library Kit, following the protocol of the manufacturer. The final libraries were evaluated on the 4,200 TapeStation (Agilent Technologies, Santa Clara, California, USA) and quantified using Qubit (Thermo Fisher Scientific, Waltham, Massachusetts, USA). Libraries were sequenced via synthesis on Miseq for paired 150-bp read lengths using the Illumina MiSeq V3 Kits (Illumina, San Diego, California, USA) (15–17).

#### Analysis of Sequences and Detection of Variants

The sequenced reads were mapped to the human mitochondria reference (NC_012920) with Burrows-Wheeler Aligner and variants were identified using the Genome Analysis Toolkit. The sequence variants were filtered using various quality parameters. For NGS technology, each template was sequenced individually; therefore, quantitative analysis of the heteroplasmic mutant load was possible by counting the number of mtDNA reads (18, 19).

#### Data and Statistical Analysis

The genetic and clinical characteristics of the seven patients with Leigh syndrome with *MT-ND3* mutation are given in Table 1. Statistical analysis was performed using SPSS version 20.0 for Windows (IBM Corporation, Armonk, New York, USA). Descriptive statistics were used including the median and range. The mutation load of mtDNA in each patient was displayed as a percentage and treated as a continuous variable for statistical analysis. The Pearson correlation coefficients (*r*) were calculated to see the correlation between two continuous variables. The statistical significance level was set at *p* < 0.05.

**Table 1:** General characteristics of Leigh syndrome with *MT-ND3* mutation (Total *N* = 7).

	**Patient 1**	**Patient 2**	**Patient 3**	**Patient 4**	**Patient 5**	**Patient 6**	**Patient 7**
Gender	Male	Female	Female	Male	Female	Female	Female
Age of first Sx (mo)	2	8	12	6.2	12	44	18
First Sx	Seizure	DD	DD	Seizure	DD	Seizure	Visual disturbance
Organ involvement							
CNS	O	O	O	O	O	O	O
Muscle weakness	O	O	X	X	X	O	X
Respiratory system	O	O	O	O	O	O	X
Cardiology	X	X	X	X	X	X	O
GI system	O	O	O	O	O	O	X
Endocrine system	O	O	O	X	O	X	X
Kidney	O	O	X	X	O	X	X
Ophthalmology	O	X	O	X	X	O	O
Ear	X	X	X	X	X	O	X
Skeletal system	X	X	O	X	X	X	O
Magnetic resonance imaging obtained					
Basal ganglia	O	O	O	O	O	O	O
Thalamus	O	O	O	O	X	X	O
Brain stem	O	O	O	O	O	O	O
Cerebral atrophy	O	O	O	O	X	X	X
Cerebellar atrophy	O	X	O	X	X	X	X
Cortex/subcortex signal abnormality	O	O	O	X	X	X	X
White matter signal abnormality	O	X	O	X	X	X	X
Magnetic resonance spectroscopy obtained					
Presence of lactate peak	O	O	O	O	X	O	X
Decreased NAA peak	O	X	X	X	X	X	X
Mitochondrial variables							
Serum lactic acidosis at diagnosis (normal range : 0.5–1.6 mmol/L)	3.3	3.7	4.2	1.5	3	3.1	6.8
Grading of serum lactic acidosis	moderate	moderate	moderate	normal	mild	mild	severe
Muscle biopsy obtained							
Light microscopic changes	non-specific	non-specific	non-specific	non-specific	Ragged red fiber	non-specific	non-specific
Electron microscopic changes							
EM-pleoconia	X	X	X	X	O	X	X
EM-megaconia	X	X	X	X	O	X	X
mtDNA mutation position	m.10191 T>C	m.10191 T>C	m.10191 T>C	m.10191 T>C	m.10191 T>C	m.10191 T>C	m.10158 T>C
Mutant load (%)	69.7	82.9	62.9	88.2	81.5	93.6	57.9
Mitochondrial cocktail treatment	O	O	O	O	O	O	O

*No., number; Sx, symptom; mo, month; CNS, central nervous system; GI, gastro-intestinal tract; DD, developmental delay or regression; NAA, N-acetylaspartate; O, yes, X, no*.

## Results

### General Characteristics

Among the seven patients with Leigh syndrome with *MT-ND3* mutation, the male-to-female ratio was 2:5. The median age at first symptom onset was 12 months and ranged from 12 to 44 months. Six of the seven patients experienced early-onset Leigh syndrome and, therefore, developed symptoms before 24 months of age. The first symptoms of the patients were primarily developmental delay/regression and seizures (three patients each). The organ involvement in the patients at the time of the last follow-up was identified. CNS symptoms were observed in 100% of the patients, followed by symptoms related to the gastrointestinal (GI) tract (*n* = 6, 85.7%) and the respiratory system (*n* = 6, 85.7%) and ophthalmological symptoms (*n* = 5, 71.4%). Normotonic or hypertonic muscle tone was observed in all the patients. Three patients had symptoms of dystonia. All the patients had gait disorder and three patients with muscle weakness could not walk at all. Among the patients, six patients were receiving enteral tube feeding, two patients underwent gastrostomy, and four patients were fed through temporary enteral tube feeding (nasogastric tube). The median age at the time of Leigh syndrome diagnosis was 22 months (range, 5–70 months). The median interval time between the development of the first symptom and Leigh syndrome diagnosis was 12 months (range, 1–44 months) (Table 1).

### Magnetic Resonance Imaging/MRS of the Patient

**Figure 1:**
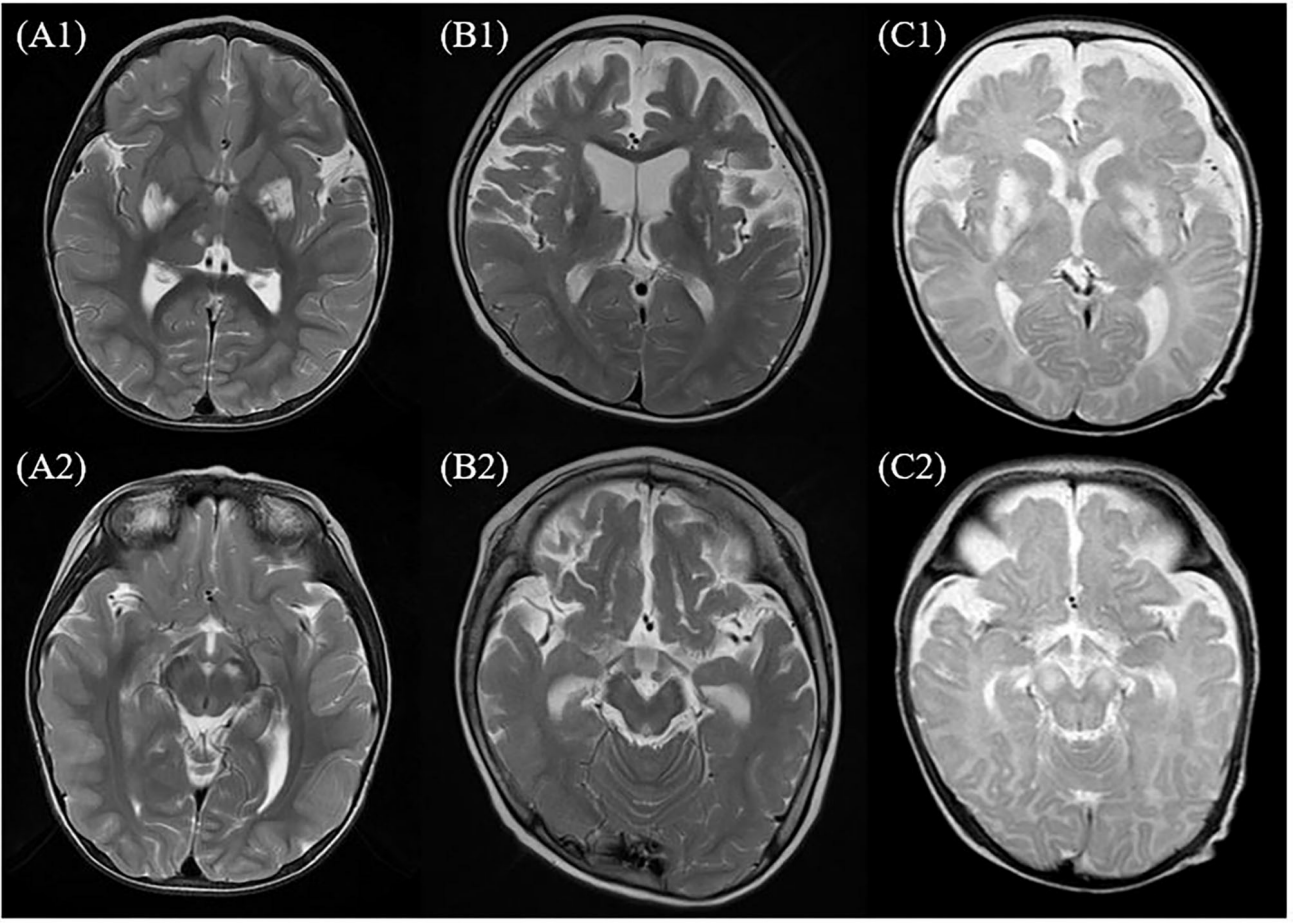
MRI of the brain findings in the patients with Leigh syndrome with *MT-ND3* mutation. This figure shows some findings from T2-weighted axial images of MRI of the brain in the patients with Leigh syndrome with *MT-ND3* mutation. **(A1,A2)** Hyperintense signal abnormalities are observed in both the basal ganglia, brainstem, and thalamus (patient 7). **(B1,B2)** Hyperintense signal abnormalities in both the basal ganglia and diffuse brain atrophy prominently at bilateral frontotemporal lobes with secondary ventricular dilatation are observed (patient 2). **(C1,C2)** Diffuse abnormal signal changes at both the basal ganglia, thalami, and brainstem are observed and diffuse brain atrophy is also observed (patient 1).

Magnetic resonance imaging was performed for all the patients. Involvement of the basal ganglia was identified as 100%, followed by the involvement of the brainstem or thalamus. MRS was performed for six of the patients presenting the m.10191T> C mutation and lactate peaks were detected in five of the six patients (Table 1 and Figure 1).

### Genetic and Mitochondrial Characteristics

**Figure 2:**
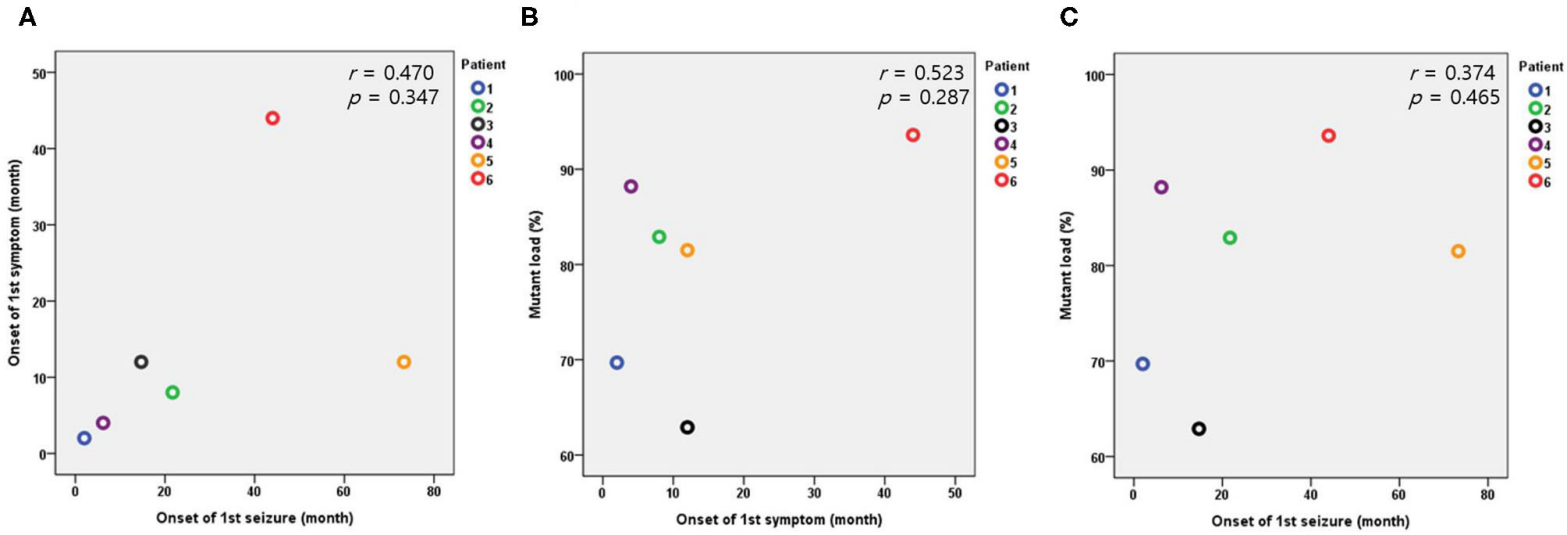
Relationships between the onset of the first symptom, first seizure, and mutant load in the patients with the m.10191T>C mutation. **(A)** Relationship between the onset of the first symptom and first seizure. **(B)** Relationship between the onset of the first symptom and mutant load. **(C)** Relationship between the onset of the first seizure and mutant load.

The median value of serum lactic acidosis at diagnosis was 3.3 and ranged from 1.5 to 6.8. The serum lactic acidosis of six of the seven patients was mildly to severely increased. No specific findings were identified from muscle biopsies using light and electron microscopy. The seven patients with Leigh syndrome with *MT-ND3* mutation were divided into the m.10191T>C and the m.10158T>C groups, according to the respective nucleotide change. The majority of the patients (*n* = 6) were in the m.10191T>C group. The median value of the mutant load was 82.5%, ranging from 57.9 to 93.6%. All the patients received mitochondrial cocktail treatment such as coenzyme, L-carnitine, and multivitamins from the time of diagnosis. No particular tendency was observed for the first symptom, first seizure onset, or mutant load. The Pearson correlation coefficient and significance level are *r* = 0.470, *p* = 0.347 in Figure 2A; *r* = 0.523, *p* = 0.287 in Figure 2B; and *r* = 0.374, *p* = 0.465 in Figure 2C, respectively (Table 1 and Figure 2).

### Epilepsy Characteristics of the Patients With Leigh Syndrome With MT-ND3 Mutation

The prevalence of epilepsy was high in Leigh syndrome with *MT-ND3* mutation (*n* = 6). In particular, patients 1, 2, 3, 4, 5, and 6 (m.10191T>C mutation) were diagnosed with epilepsy and treated accordingly. The median age at first seizure was 18.2 months and ranged from 2 to 73.3 months. The seizure types consisted of two generalized tonic seizures, two focal seizures, one focal-to-secondary generalized seizure, and one mixed-type seizure. Three of the six patients with epilepsy had a history of status epilepticus. In the EEG findings at the last follow-up, the EEG patterns of Lennox–Gastaut syndrome (LGS), such as multifocal sharp, generalized sharp and slow wave (GSSW) discharge, and generalized paroxysmal fast activity (GPFA), were observed in three of the six patients with epilepsy (50% of the m.10191T>C mutation group, patient numbers 1, 2, and 3). These three patients had no history of infantile spasm. Five of the six patients with epilepsy were taking multiple antiseizure medications (ASMs) and two patients had attempted the ketogenic diet (KD) (lipid: nonlipid ratio: 4:1). No patients experienced serious side effects due to the ASMs, but the KD was discontinued early due to poor oral intake and GI problems in the two patients. The current seizure status compared to the initial seizure frequency was investigated and the two seizure-free patients were identified. One patient experienced > 90% reduction in seizure frequency a second patient experienced 50–90% reduction in seizure frequency, a third patient experienced < 50% reduction in seizure frequency, and a fourth patient experienced no change. The current status of deterioration was also measured and progressive deterioration was observed in four of the seven patients of Leigh syndrome with *MT-ND3* mutation and the remaining three patients had a static status and no longer experienced progressive deterioration (Table 2).

**Table 2:** Epilepsy characteristics of Leigh syndrome with *MT-ND3* mutation (Total *N* = 7).

	**Patient 1**	**Patient 2**	**Patient 3**	**Patient 4**	**Patient 5**	**Patient 6**	**Patient 7**
Epilepsy	LGS	LGS	LGS	Focal epilepsy	Focal epilepsy	Focal epilepsy	none
Age of first seizure (mo)	2.0	21.7	14.7	6.2	73.3	44.0	-
Seizure type	GT with multiple type seizure	GT with multiple type seizure	GT with multiple type seizure	Focal	Mixed type	Focal	-
Seizure frequency (per month)	15	2	1	1	30	10	-
Status epilepticus	X	X	O	O	O	X	-
EEG of last f/u	S&D BG, multifocal sharp, GSSW	S&D BG, multifocal sharp, GSSW	S&D BG, multifocal sharp, GSSW, GPFA	S&D BG (asymmetric focal slowing)	S&D BG (asymmetric focal slowing)	S&D BG (asymmetric focal slowing)	S&D BG
Current ASM	PB, PHT, CLZ, LMT, OXC	VGB, VPA	VPA, ZNS, RUF, CLB, PTH	discontinued	VPA, PB, CLZ, ZNS	CBZ, PB, ZNS	-
KD	O	X	X	O	X	X	-
Current status of seizure (compared to initial seizure frequency)	50-90% reduction of seizure frequency	free	>90% reduction of seizure frequency	free	<50% reduction of seizure frequency	No change of seizure frequency	no history of seizures
Current status of deterioration	static	static	• progressive • deteriorating	• progressive • deteriorating	• progressive • deteriorating	• progressive • deteriorating	static

*No., number; mo, month; ASM, anti-seizure medication; KD, ketogenic diet; LGS, Lennox-Gastaut syndrome; S&D BG, slow and disorganized background; GSSW, generalized sharp and slow wave discharge; GPFA, generalized paroxysmal fast activity; VPA, valproic acid; PB, phenobarbital; CLZ, clonazepam; ZNS, zonisamide; PTH, phenytoin; LMT, lamotrigine; OXC, oxcarbazepine; VGB, vigabatrin; RUF, rufinamide; CLB, clobazam; CBZ, carbamazepine*.

## Discussion

Epilepsy is a major phenotype of mitochondrial diseases (20). In particular, Leigh syndrome is the most common mitochondrial disease in childhood and high epilepsy morbidity has been reported (21). Among the various presentations of mtDNA-associated Leigh syndrome, several studies have reported a specific gene related to epilepsy. Some studies have found that m.10191T>C, a major nucleotide change in *MT-ND3* gene, is strongly related to epilepsy (7, 21). In this study, the six patients with the m.10191T>C mutation were associated with epilepsy, suggesting that this mutation is important in Leigh syndrome with *MT-ND3* mutation. Additionally, the results of this study suggested that even the same *MT-ND3* mutation may have different phenotypes depending on the location of the point mutation. These results are supported by two important studies. Nesbitt et al. reviewed the clinical spectra of 16 patients with the m.10191T>C mutation from 14 references. This study does not describe a close relationship between epilepsy and m.10191T>C. However, after reviewing the 14 case reports used in this study, seizures were identified in 12 of the 16 (75%) patients with the m.10191T>C mutation. Their EEG findings were unknown, but most were found to be strongly associated with epilepsy. Rebecca et al. added several cases to those reviewed by Nesbitt et al. and modified the clinical features of the patients with the m.10191T>C mutation. In this study, seizures were identified in 15 out of 22 (68.2%) patients with the m.10191T>C mutation (6, 8). In addition, a recent study by Li et al. reviewed 28 patients with the m.10191T>C mutation from 23 references. Among them, 24 (85.7%) patients with the m.10191T>C mutation had epilepsy as their phenotype. Therefore, this study states that the m.10191T>C mutation may be strongly associated with epilepsy and these findings are consistent with those of this study (7). The value of this study lies in the fact that its findings are consistent with those of the previous studies and that it undertakes a more focused analysis of the characteristics of epilepsy in Leigh syndrome with the m.10191T>C mutations.

Lennox–Gastaut syndrome is rare and is one of the most severe forms of epilepsy with childhood onset. Epileptic discharges, known as GSSW complexes and GPFA in EEG, multiple types of seizures, and severe intellectual impairments are the diagnostic triad of LGS. LGS is one of the most complex epileptic disorders to manage, both for the pediatric neurologists and epilepsy specialists (22, 23). In a study by Lee et al., of the 372 patients with a mitochondrial disease, 40 patients were diagnosed with LGS, which represented about 10% of their study cohort (23). To the best of our knowledge, there are no previous studies on the relationship between LGS and mitochondrial disease or Leigh syndrome. Therefore, genotype–phenotype correlations are necessary to establish a cohort and analyze these relationships. In this study, 50% of the six patients with the m.10191T>C mutation and epilepsy had LGS. Although this study included a small cohort, considering that Leigh syndrome with the m.10191T>C mutation and with LGS are both the rare diseases, LGS may be frequently associated with the epilepsy phenotype in the m.10191T>C mutation. If the m.10191T>C mutation has features that are often accompanied by LGS, it may be possible to control the disease by providing intensive LGS treatment to these patients. To the best of our knowledge, no previous studies support this, but we will continue to explore the relationship between LGS and the m.10191T>C mutation in future studies.

Heteroplasmy is an important feature of mtDNA-associated Leigh syndrome. Heteroplasmy rarely affects the type and severity of phenotypes. Within the same mutation, heteroplasmy is expected to be associated with disease severity, reflected by the specific phenotypic presentation or the time of symptom onset (4, 24). However, in this study, no correlation was identified between heteroplasmy and the onset of the first symptom or seizure. In addition, among the patients with *MT-ND3* mutation, no significant difference in heteroplasmy was observed between patients with or without LGS. This may be because the total number of patients in this study was relatively small. In addition, the possibility of other factors and of heteroplasmy acting upon the onset of symptoms can also be considered. Also, although not measured in this study, heteroplasmy in brain may be different than in the blood leukocytes measured and might show a closer relationship to the neurologic presentations (7).

Two patients started the KD; one had LGS and the other had not LGS. However, both the patients discontinued the KD after 3 months due to poor oral intake and severe GI problems. This suggested possible intolerance for KDs in the patients with the m.10191T>C mutation, but a larger cohort is necessary to establish a firm conclusion. Kang et al. reported the safety and effective application of the KD in mitochondrial respiratory chain complex defects. In this study, four patients had mitochondrial disease with LGS. The KD was effective for seizure reduction in all the patients. However, the maintenance duration of the KD varied, with one patient discontinuing the KD after 3 months due to severe hypoglycemia and the other three patients maintained the KD for 6, 12, and 24 months. Two patients became seizure free (25). However, this treatment was not conducted through gene-based targeted therapy. Therefore, with the results of this study, the effect and stability of the KD may not be suitable for patients with the m.10191T>C mutation. At present, there are no studies with respect to the relationship between the KD and *MT-ND3* or the m.10191T>C mutations. Genotype-based KD studies should be conducted in the future (26).

Valproic acid (VPA) is a broad-spectrum antiepileptic drug that is widely used as a first-line treatment for most types of epilepsy. In particular, patients with LGS are considered for first-line treatment (27). However, because VPA can induce mitochondrial toxicity through the inhibition and subsequent decreased activity of mitochondrial complexes I and IV, it is contraindicated for patients with a mitochondrial disease (28–30). However, in this study, VPA was used in three of the six patients with the m.10191T>C mutation and an improvement in seizure frequency was observed. Two of the three patients were diagnosed with LGS. VPA therapy may be effective in treating epilepsy depending on the clinical situation and phenotype and may not cause a contraindication. In particular, patients with the m.10191T>C mutation have mtDNA-associated Leigh syndrome with a high morbidity of epilepsy that may be accompanied by refractory epilepsy such as LGS. In such situations, mitochondrial dysfunction may be diagnosed later as the etiology of epilepsy. Therefore, the careful application of VPA may assist in controlling adverse effects or mitochondrial toxicity.

In conclusion, the results of this study indicate a strong association between epilepsy and Leigh syndrome with *MT-ND3* mutation, particularly the m.10191T>C mutation. Additionally, there is a possibility of an association between the epilepsy phenotype of the m.10191T>C mutation and LGS. Due to the characteristics of rare disease, there is a limitation in that this study could not be conducted on a large homogeneous patient group. Therefore, a follow-up study or meta-analysis with a large number of patients is needed in the future. This study explored the importance of specific point mutations such as the m.10191T>C mutation, although *MT-ND3* mutation is important for genotype–phenotype correlation. Understanding the characteristics of Leigh syndrome with *MT-ND3* mutation may assist in predicting disease progression and improving patient survival through timely treatment. This understanding could be the first step in finding biomarkers for the development of new treatments for Leigh syndrome (31).

## Data Availability Statement

The original contributions presented in the study are included in the article/supplementary material, further inquiries can be directed to the corresponding author.

## Ethics Statement

This study was approved by the Institutional Review Board of Gangnam Severance Hospital, Yonsei University College of Medicine (3-2017-0168). Written informed consent to participate in this study was provided by the participants' legal guardian/next of kin.

## Author Contributions

Y-ML conceptualized and designed the study, coordinated and supervised data collection, and critically reviewed and revised the manuscript. J-HN designed the data collection instruments, collected data, carried out the initial analyses, drafted the initial manuscript and revised the manuscript. ML and CL coordinated and supervised data collection. All authors approved the final manuscript as submitted and agree to be accountable for the content of the work.

## Funding

This study was supported by a grant from the Korea Health Technology R&D Project through the Korea Health Industry Development Institute (KHIDI), funded by the Ministry of Health and Family Welfare, Republic of Korea (Grant Numbers: 2018-31-0425/HI18C1166020018, 2018-31-1061/HI18C1166020019, and 2019-31-1183/HI18C1166020020).

## Conflict of Interest

The authors declare that the research was conducted in the absence of any commercial or financial relationships that could be construed as a potential conflict of interest.

## Publisher's Note

All claims expressed in this article are solely those of the authors and do not necessarily represent those of their affiliated organizations, or those of the publisher, the editors and the reviewers. Any product that may be evaluated in this article, or claim that may be made by its manufacturer, is not guaranteed or endorsed by the publisher.
